# To Banish Rats

**Published:** 1874-12

**Authors:** 


To Banish Rats.—A good way to banish rats is to plant asphodel near the barn or stable where they are, or put some in their holes. Rats have such an aversion for this plant that they will quit the premises where it is. If they are in drains or in cellars, scatter sulphate of iron (copperas) in their runs. The copperas should not be dissolved. It is one of our best and cheapest disinfectants. The sulphuric acid in the copperas burns their feet as soon as it dissolves, and they leave in a short time, without dying. This will be appreciated by every housekeeper who has had to endure the stench of a dead rat.

